# {2-[(η^5^-Cyclo­penta­dien­yl)diphenyl­meth­yl]-1*H*-imidazolido-κ*N*}bis­(*N*,*N*-diethyl­amido)­titanium(IV)

**DOI:** 10.1107/S1600536811012396

**Published:** 2011-04-07

**Authors:** Xianfeng Cai, Yingying Xu, Wanli Nie, Maxim V. Borzov

**Affiliations:** a Key Laboratory of Synthetic and Natural Chemistry of the Ministry of Education, College of Chemistry and Material Science, The North-West University of Xi’an, Taibai Bei Avenue 229, Xi’an 710069, Shaanxi Province, People’s Republic of China

## Abstract

The chemically achiral title mol­ecule, [Ti(C_4_H_10_N)_2_(C_21_H_16_N_2_)], crystallizes in the chiral space group *P*2_1_. All three N atoms coordinating to the Ti^IV^ atom adopt planar environments [sums of valence angles = 359.5 (6), 360.0 (7) and 360.0 (6)°], which is indicative of *p*π–*d*π donation from all of these N atoms to the metal and, thus, of the formal 18 e^−^ nature of the complex. The overall coordination about the Ti^IV^ atom is distorted tetra­hedral, assuming the cyclo­penta­dienyl ring occupies one coordination site. The Ti—N_imidazole_ amide-type bond is longer by approximately 0.16 Å than the other two Ti—N_amide_ bonds.

## Related literature

For structural parameters of η^5^-CpTi-tris­(*sec*-amido)-type complexes, see: Rhodes *et al.* (2002 ▶); Li *et al.* (2003 ▶); Seo *et al.* (2001 ▶); Kunz *et al.* (2001 ▶, 2002 ▶); Carpenetti *et al.* (1996 ▶); Bertolasi *et al.* (2007 ▶); Wu *et al.* (2006 ▶); Cano *et al.* (2005 ▶); Martin *et al.* (1994 ▶). For two related Ti^IV^ complexes, see: Wang *et al.* (2009 ▶). For the structural parameters of 1*H*-imidazol(in)-2-yl side-chain functionalized cyclo­penta­dienes and their Li, Ti, and Zr complexes, see: Krut’ko *et al.* (2006 ▶); Nie *et al.* (2008 ▶); Sun *et al.* (2009 ▶, 2010 ▶); Ge *et al.* (2010 ▶). For synthetic details, see: Curtis & Brown (1980 ▶); Bürger & Dämmen (1974 ▶); Bradley & Thomas (1960 ▶); Chajara & Ottosson (2004 ▶); Armarego & Perrin (1997 ▶). For a description of the Cambridge Structural Database, see: Allen (2002 ▶).
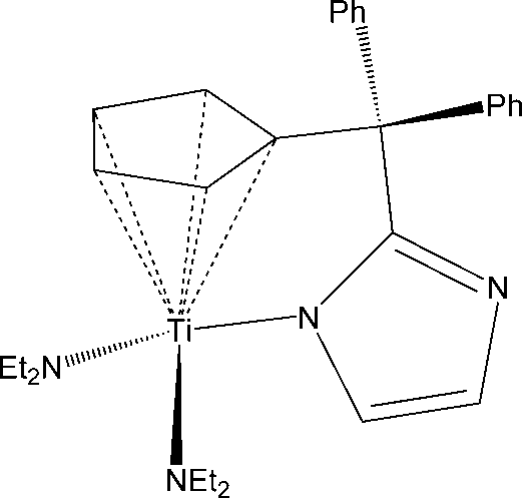


## Experimental

### 

#### Crystal data

[Ti(C_4_H_10_N)_2_(C_21_H_16_N_2_)]
*M* *_r_* = 488.52
Monoclinic, 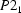

*a* = 8.6495 (6) Å
*b* = 17.9486 (12) Å
*c* = 9.1130 (6) Å
β = 110.603 (1)°
*V* = 1324.27 (15) Å^3^
*Z* = 2
Mo *K*α radiation
μ = 0.35 mm^−1^
*T* = 296 K
0.35 × 0.23 × 0.08 mm

#### Data collection

Bruker SMART APEXII diffractometer
Absorption correction: multi-scan (*SADABS*; Sheldrick, 1996 ▶) *T*_min_ = 0.888, *T*_max_ = 0.973
7145 measured reflections
4906 independent reflections
4031 reflections with *I* > 2σ(*I*)
*R*_int_ = 0.022

#### Refinement

*R*[*F* ^2^ > 2σ(*F* ^2^)] = 0.040
*wR*(*F* ^2^) = 0.102
*S* = 0.99
4906 reflections
311 parameters
1 restraint
H-atom parameters constrained
Δρ_max_ = 0.21 e Å^−3^
Δρ_min_ = −0.21 e Å^−3^
Absolute structure: Flack (1983 ▶), 2209 Friedel pairs
Flack parameter: 0.02 (3)

### 

Data collection: *APEX2* (Bruker, 2007 ▶); cell refinement: *SAINT* (Bruker, 2007 ▶); data reduction: *SAINT*; program(s) used to solve structure: *SHELXS97* (Sheldrick, 2008 ▶); program(s) used to refine structure: *SHELXL97* (Sheldrick, 2008 ▶); molecular graphics: *OLEX2* (Dolomanov *et al.*, 2009 ▶); software used to prepare material for publication: *SHELXTL* (Sheldrick, 2008 ▶) and *OLEX2*.

## Supplementary Material

Crystal structure: contains datablocks I, global. DOI: 10.1107/S1600536811012396/wm2469sup1.cif

Structure factors: contains datablocks I. DOI: 10.1107/S1600536811012396/wm2469Isup2.hkl

Additional supplementary materials:  crystallographic information; 3D view; checkCIF report

## Figures and Tables

**Fig. 1.**
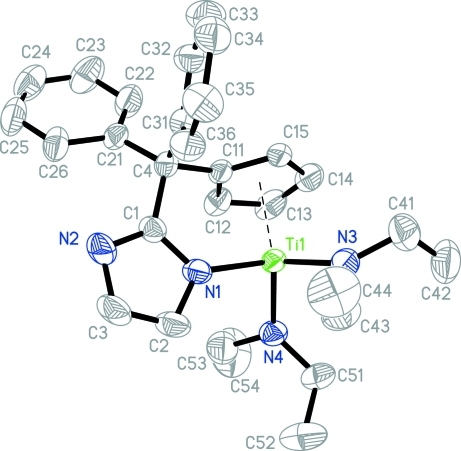
The molecule of compound (I) in the asymmetric unit with the labelling scheme. Displacement ellipsoids are shown at the 50% probability level. All H atoms have been omitted for clarity.

## supplementary crystallographic information

### Comment

Cyclopentadienes (Cp-s) with pendant 1*H*-imidazol(in)-2-yl side-chain functional groups, their mono- and di-Li salts, and group 4 transition metal complexes of general type [η^5^-Cp-(C_1_ or C_2_)-imidazol(in)e)-κ*N*]-*M*^IV^*L*_n_ [*M* = Ti, Zr; *L*_n_ = Cl_3_, (N*R*_2_)_2_] have been described previously (Krut'ko *et al.*, 2006; Nie *et al.*, 2008; Sun *et al.*, 2009; Wang *et al.*, 2009; Ge *et al.*, 2010; Sun *et al.*, 2010). The present contribution reports the structural investigation of the closest analogue of two Ti^IV^ 18e^-^ (η^5^-Cp)tris(*sec*-amido)- type complexes described recently by Wang *et al.* (2009).

The title complex, [Ti(C_4_H_10_N)_2_(C_21_H_16_N_2_)], (I), was prepared by treatment of 2-[(cyclopentadienyl)diphenylmethyl]-1*H*-imidazole, C_21_H_18_N_2_, (II), with Ti(NEt_2_)_4_ in toluene by an analogy to what described in Wang *et al.* (2009) (see Experimental for more details). Of interest, despite its molecule is chemically achiral, (I) crystallizes in the chiral space group *P*2_1_. The Ti^IV^ is in a distorted tetrahedral environment (assuming the Cp-ring occupies one coordination site), with all three N-atoms coordinating to the Ti-centre adopting planar environments [valent angles sums 359.5 (6), 360.0 (7), and 360.0 (6)°, respectively] what is indicative of the *p*π–*d*π donation from all of these N-atoms to the metal and, thus, of the formal 18e^-^ nature of the complex. The Ti—N_imidazole_ amido-type bond is approximately by 0.16 Å longer than the other two Ti—N amido-bonds, presumably due to the ridgid bridge constraint and/or certain electronic effects (e.g. involvment of the N_imidazole_*p*-AO into the aromatic system of the heterocycle ring).

The bond lengths and angles at the Ti atom are close to those reported by Wang *et al.* (2009). The distance of the r.m.s. plane (PL1) of the Cp ring (C11–C15) to the Ti atom is 2.057 Å. The Ti atom only slightly deviates from the r.m.s. plane (PL2) of the imidazole C1/N1/C2/C3/N2 ring by 0.201 (5) Å, with the PL1–PL2 angle being 101.58 (14)°.

Analysis of the Cambridge Structural Database (version 5.27, release: May 2009) (Allen, 2002) reveals 16 structurally characterized Ti complexes of similar η^5^-CpTi-tris(*sec*-amido) types (22 independent fragments) (Rhodes *et al.*, 2002; Li *et al.*, 2003; Seo *et al.*, 2001; Kunz *et al.*, 2002; Carpenetti *et al.*, 1996; Kunz *et al.*, 2001; Bertolasi *et al.*, 2007; Wu *et al.*, 2006; Cano *et al.*, 2005; Martin *et al.*, 1994). Noteworthy, that except of η^5^-Cp*-Ti(NMe_2_)_3_ (Martin *et al.*, 1994) all these complexes as in case of compound (I) contain, at least, one amido-functionality linked to the Cp-ring with a flexible bridge.

### Experimental

All operations were performed under Ar atmosphere in conventional glassware or in all-sealed evacuated glass vessels with application of a high-vacuum line (the residual pressure of non-condensable gases within 1.5–1.0×10 ^-3^ Torr; 1 Torr = 133 Pa). 5-(Diphenylmethylidene)cyclopenta-1,3-diene (6,6-diphenylfulvene) was prepared as described by Chajara & Ottosson (2004). Ti(NEt_2_)_4_ was prepared as described earlier by Bürger & Dämmen (1974); Bradley & Thomas (1960). 1-Diethoxymethyl-1*H*-imidazole and its 2-lithiated derivative were prepared as described by Curtis & Brown (1980). All other chemicals were commercially available and purified by conventional methods (Armarego & Perrin, 1997). Solvents were purified by distillation over sodium benzophenoneketyl (diethyl ether, THF), Na—K alloy (toluene, hexane, benzene), and CaH_2_ (chloroform). The deuterated solvent (C_6_D_6_) was dried similarly. Compound (I) was prepared in a full analogy to what was described by Wang *et al.* (2009). The NMR spectra were recorded on a Varian INOVA-400 instrument. For ^1^H spectrum, the TMS (δ_H_ = 0.00 and δ_C_ = 0.0) resonance was used as an internal reference standard. ^1^H NMR (298 K, C_6_D_6_): δ = 0.70 (virt. t, an X-part of an ABX_3_ spin system, 12 H, ^3^*J*_AX_ = ^3^*J*_BX_ = 6.7 Hz, NCH_2_C*H*_3_), 3.12, 3.46 (both virt. dq, an AB-part of an ABX_3_ spin system, 4 H + 4H, ^3^*J*_AX_ = ^3^*J*_BX_ = 6.7 Hz, ^2^*J*_AB_ = 14.0 Hz, NC*H*_2_CH_3_), 5.74 (unresolved m, 4 H, CH in Cp), 7.04, 7.15, 7.86 (all m, *p*-, *m*-, and *o*-CH in Ph, in respective order), 7.21, 7.55 (an AB spin system, 1 H + 1 H, ^2^*J*_AB_ = 1.2 Hz, imidazole ring protons).

A crystal of (I) suitable for X-ray diffraction analysis was picked up from the isolated material and mounted inside a Lindemann glass capillary (diameter 0.5 mm; N_2_-filled glove-box).

### Refinement

H atoms were treated as riding atoms with distances C—H = 0.96 (CH_3_), 0.97 (CH_2_), 0.93 Å (C_Ar_H), and *U*_iso_(H) = 1.5 *U*_eq_(C), 1.2 *U*_eq_(C), and 1.2 *U*_eq_(C), respectively.

### Crystal data

**Table:**

[Ti(C_4_H_10_N)_2_(C_21_H_16_N_2_)]	*F*(000) = 520
*M**_r_* = 488.52	*D*_x_ = 1.225 Mg m^−^^3^
Monoclinic, *P*2_1_	Mo *K*α radiation, λ = 0.71073 Å
Hall symbol: P 2yb	Cell parameters from 5258 reflections
*a* = 8.6495 (6) Å	θ = 2.6–28.2°
*b* = 17.9486 (12) Å	µ = 0.35 mm^−^^1^
*c* = 9.1130 (6) Å	*T* = 296 K
β = 110.603 (1)°	Block, brown
*V* = 1324.27 (15) Å^3^	0.35 × 0.23 × 0.08 mm
*Z* = 2	

### Data collection

**Table:**

Bruker SMART APEXII diffractometer	4906 independent reflections
Radiation source: fine-focus sealed tube	4031 reflections with *I* > 2σ(*I*)
graphite	*R*_int_ = 0.022
Detector resolution: 8.333 pixels mm^-1^	θ_max_ = 26.0°, θ_min_ = 2.3°
φ and ω scans	*h* = −8→10
Absorption correction: multi-scan (*SADABS*; Sheldrick, 1996)	*k* = −22→22
*T*_min_ = 0.888, *T*_max_ = 0.973	*l* = −11→9
7145 measured reflections	

### Refinement

**Table:**

Refinement on *F*^2^	Secondary atom site location: difference Fourier map
Least-squares matrix: full	Hydrogen site location: inferred from neighbouring sites
*R*[*F*^2^ > 2σ(*F*^2^)] = 0.040	H-atom parameters constrained
*wR*(*F*^2^) = 0.102	*w* = 1/[σ^2^(*F*_o_^2^) + (0.0594*P*)^2^] where *P* = (*F*_o_^2^ + 2*F*_c_^2^)/3
*S* = 0.99	(Δ/σ)_max_ < 0.001
4906 reflections	Δρ_max_ = 0.21 e Å^−^^3^
311 parameters	Δρ_min_ = −0.21 e Å^−^^3^
1 restraint	Absolute structure: Flack (1983), 2209 Friedel pairs
Primary atom site location: structure-invariant direct methods	Flack parameter: 0.02 (3)

### Special details

**Table:**

Geometry. All esds (except the esd in the dihedral angle between two l.s. planes) are estimated using the full covariance matrix. The cell esds are taken into account individually in the estimation of esds in distances, angles and torsion angles; correlations between esds in cell parameters are only used when they are defined by crystal symmetry. An approximate (isotropic) treatment of cell esds is used for estimating esds involving l.s. planes.
Refinement. Refinement of F^2^ against ALL reflections. The weighted R-factor wR and goodness of fit S are based on F^2^, conventional R-factors R are based on F, with F set to zero for negative F^2^. The threshold expression of F^2^ > 2sigma(F^2^) is used only for calculating R-factors(gt) etc. and is not relevant to the choice of reflections for refinement. R-factors based on F^2^ are statistically about twice as large as those based on F, and R- factors based on ALL data will be even larger.

### Fractional atomic coordinates and isotropic or equivalent isotropic displacement parameters (Å^2^)

**Table:**

	*x*	*y*	*z*	*U*_iso_*/*U*_eq_	
Ti1	0.80413 (5)	0.23594 (2)	0.88291 (5)	0.03379 (13)	
N1	0.5871 (3)	0.24357 (15)	0.9268 (3)	0.0402 (5)	
N2	0.3929 (3)	0.21126 (16)	1.0254 (4)	0.0546 (8)	
N3	0.7642 (3)	0.23863 (19)	0.6661 (3)	0.0484 (5)	
N4	0.8943 (3)	0.33169 (14)	0.9552 (3)	0.0423 (6)	
C1	0.5246 (3)	0.19024 (16)	0.9950 (4)	0.0371 (6)	
C2	0.4852 (4)	0.30452 (18)	0.9156 (4)	0.0514 (9)	
H2	0.4947	0.3511	0.8747	0.062*	
C3	0.3696 (5)	0.2835 (2)	0.9755 (5)	0.0592 (10)	
H3	0.2854	0.3140	0.9818	0.071*	
C4	0.5952 (3)	0.11126 (15)	1.0097 (3)	0.0347 (6)	
C11	0.7649 (3)	0.12130 (15)	0.9937 (3)	0.0354 (6)	
C12	0.8975 (3)	0.15962 (18)	1.1051 (4)	0.0433 (7)	
H12	0.8977	0.1779	1.2007	0.052*	
C13	1.0270 (4)	0.1656 (2)	1.0498 (4)	0.0547 (9)	
H13	1.1282	0.1885	1.1008	0.066*	
C14	0.9777 (4)	0.13112 (19)	0.9038 (5)	0.0572 (10)	
H14	1.0407	0.1274	0.8399	0.069*	
C15	0.8150 (4)	0.10237 (16)	0.8682 (4)	0.0455 (8)	
H15	0.7537	0.0761	0.7787	0.055*	
C21	0.6162 (3)	0.07332 (17)	1.1669 (3)	0.0390 (7)	
C22	0.7484 (4)	0.0259 (2)	1.2372 (4)	0.0585 (9)	
H22	0.8285	0.0199	1.1918	0.070*	
C23	0.7638 (6)	−0.0125 (3)	1.3726 (5)	0.0729 (13)	
H23	0.8511	−0.0455	1.4150	0.087*	
C24	0.6505 (5)	−0.0021 (2)	1.4449 (4)	0.0674 (11)	
H24	0.6626	−0.0266	1.5382	0.081*	
C25	0.5211 (5)	0.0442 (2)	1.3793 (4)	0.0667 (11)	
H25	0.4436	0.0510	1.4275	0.080*	
C26	0.5025 (4)	0.0819 (2)	1.2406 (4)	0.0519 (8)	
H26	0.4123	0.1132	1.1969	0.062*	
C31	0.4818 (3)	0.06271 (16)	0.8743 (3)	0.0371 (6)	
C32	0.5116 (4)	−0.01293 (19)	0.8684 (4)	0.0516 (8)	
H32	0.6011	−0.0342	0.9466	0.062*	
C33	0.4118 (5)	−0.0571 (2)	0.7495 (4)	0.0639 (10)	
H33	0.4340	−0.1078	0.7485	0.077*	
C34	0.2792 (5)	−0.0267 (2)	0.6321 (5)	0.0609 (11)	
H34	0.2116	−0.0565	0.5514	0.073*	
C35	0.2480 (5)	0.0478 (2)	0.6356 (4)	0.0611 (9)	
H35	0.1584	0.0688	0.5570	0.073*	
C36	0.3485 (4)	0.09227 (19)	0.7552 (4)	0.0492 (8)	
H36	0.3259	0.1429	0.7555	0.059*	
C41	0.8291 (5)	0.2021 (2)	0.5546 (5)	0.0678 (10)	
H41A	0.7439	0.2014	0.4514	0.081*	
H41B	0.8578	0.1509	0.5870	0.081*	
C42	0.9794 (5)	0.2417 (4)	0.5458 (5)	0.0835 (12)	
H42A	1.0255	0.2134	0.4818	0.125*	
H42B	1.0600	0.2467	0.6493	0.125*	
H42C	0.9482	0.2902	0.5006	0.125*	
C43	0.6389 (5)	0.2954 (2)	0.5932 (5)	0.0649 (10)	
H43A	0.6786	0.3270	0.5276	0.078*	
H43B	0.6253	0.3264	0.6749	0.078*	
C44	0.4723 (6)	0.2641 (3)	0.4946 (6)	0.0979 (16)	
H44A	0.4842	0.2335	0.4128	0.147*	
H44B	0.3977	0.3043	0.4490	0.147*	
H44C	0.4293	0.2347	0.5594	0.147*	
C51	0.9457 (4)	0.38919 (18)	0.8705 (4)	0.0523 (8)	
H51A	0.9370	0.3696	0.7686	0.063*	
H51B	1.0612	0.4004	0.9268	0.063*	
C52	0.8487 (5)	0.4608 (2)	0.8464 (6)	0.0828 (13)	
H52A	0.8870	0.4945	0.7848	0.124*	
H52B	0.8637	0.4830	0.9463	0.124*	
H52C	0.7336	0.4504	0.7927	0.124*	
C53	0.9123 (5)	0.3530 (2)	1.1150 (5)	0.0635 (10)	
H53A	0.8673	0.3134	1.1605	0.076*	
H53B	0.8463	0.3973	1.1103	0.076*	
C54	1.0848 (7)	0.3683 (4)	1.2218 (6)	0.1085 (19)	
H54A	1.1261	0.4116	1.1858	0.163*	
H54B	1.1535	0.3263	1.2223	0.163*	
H54C	1.0856	0.3770	1.3261	0.163*	

### Atomic displacement parameters (Å^2^)

**Table:**

	*U*^11^	*U*^22^	*U*^33^	*U*^12^	*U*^13^	*U*^23^
Ti1	0.0344 (2)	0.0293 (2)	0.0416 (2)	0.0000 (2)	0.01819 (18)	0.0026 (3)
N1	0.0359 (10)	0.0304 (13)	0.0572 (14)	0.0000 (12)	0.0199 (10)	−0.0029 (14)
N2	0.0435 (14)	0.0494 (18)	0.084 (2)	0.0053 (11)	0.0391 (15)	−0.0057 (14)
N3	0.0594 (13)	0.0446 (14)	0.0430 (12)	−0.0077 (17)	0.0203 (10)	−0.0006 (16)
N4	0.0415 (14)	0.0349 (14)	0.0521 (16)	−0.0049 (11)	0.0186 (13)	−0.0021 (12)
C1	0.0321 (14)	0.0349 (17)	0.0459 (17)	−0.0004 (12)	0.0156 (13)	−0.0050 (13)
C2	0.0457 (18)	0.0329 (18)	0.077 (3)	0.0088 (14)	0.0237 (18)	−0.0003 (16)
C3	0.0466 (19)	0.047 (2)	0.092 (3)	0.0143 (16)	0.035 (2)	−0.0057 (19)
C4	0.0321 (14)	0.0338 (15)	0.0425 (16)	−0.0008 (11)	0.0186 (12)	0.0008 (12)
C11	0.0349 (14)	0.0314 (15)	0.0456 (17)	0.0049 (11)	0.0212 (13)	0.0080 (12)
C12	0.0340 (15)	0.0463 (18)	0.0485 (17)	0.0035 (14)	0.0133 (13)	0.0138 (15)
C13	0.0332 (15)	0.054 (2)	0.078 (2)	0.0061 (15)	0.0215 (16)	0.024 (2)
C14	0.0511 (19)	0.045 (2)	0.095 (3)	0.0150 (16)	0.050 (2)	0.0195 (19)
C15	0.0584 (19)	0.0292 (16)	0.065 (2)	0.0063 (14)	0.0414 (17)	0.0053 (14)
C21	0.0392 (15)	0.0381 (16)	0.0440 (17)	−0.0108 (13)	0.0199 (13)	−0.0018 (13)
C22	0.060 (2)	0.061 (2)	0.065 (2)	0.0084 (18)	0.0344 (19)	0.0193 (18)
C23	0.071 (3)	0.078 (3)	0.067 (3)	0.002 (2)	0.022 (2)	0.033 (2)
C24	0.078 (3)	0.075 (3)	0.049 (2)	−0.017 (2)	0.022 (2)	0.0128 (19)
C25	0.074 (3)	0.083 (3)	0.059 (2)	−0.024 (2)	0.042 (2)	−0.005 (2)
C26	0.0480 (18)	0.064 (2)	0.0507 (19)	−0.0111 (16)	0.0264 (16)	−0.0032 (16)
C31	0.0409 (15)	0.0345 (16)	0.0434 (16)	−0.0025 (12)	0.0242 (13)	0.0002 (12)
C32	0.063 (2)	0.0382 (18)	0.053 (2)	0.0042 (15)	0.0196 (17)	0.0009 (15)
C33	0.085 (3)	0.043 (2)	0.063 (2)	−0.0104 (19)	0.025 (2)	−0.0089 (18)
C34	0.062 (2)	0.065 (3)	0.057 (2)	−0.018 (2)	0.023 (2)	−0.019 (2)
C35	0.0541 (19)	0.066 (3)	0.057 (2)	0.0007 (18)	0.0109 (17)	−0.0064 (19)
C36	0.0462 (17)	0.0469 (19)	0.0514 (19)	0.0036 (15)	0.0131 (15)	0.0002 (16)
C41	0.085 (3)	0.070 (2)	0.054 (2)	−0.007 (2)	0.032 (2)	−0.0125 (18)
C42	0.093 (3)	0.101 (3)	0.077 (2)	−0.017 (3)	0.055 (2)	−0.005 (3)
C43	0.078 (2)	0.052 (2)	0.058 (2)	−0.0039 (19)	0.0161 (19)	0.0087 (18)
C44	0.074 (3)	0.113 (4)	0.081 (3)	0.001 (3)	−0.005 (2)	0.008 (3)
C51	0.0487 (18)	0.0391 (18)	0.066 (2)	−0.0058 (15)	0.0161 (17)	0.0060 (16)
C52	0.074 (3)	0.045 (2)	0.118 (4)	0.005 (2)	0.019 (3)	0.016 (2)
C53	0.071 (3)	0.060 (2)	0.065 (2)	−0.016 (2)	0.030 (2)	−0.014 (2)
C54	0.093 (4)	0.151 (6)	0.067 (3)	−0.030 (3)	0.009 (3)	−0.030 (3)

### Geometric parameters (Å, °)

**Table:**

Ti1—N3	1.883 (2)	C24—H24	0.9300
Ti1—N4	1.906 (2)	C25—C26	1.392 (5)
Ti1—N1	2.057 (2)	C25—H25	0.9300
Ti1—C12	2.341 (3)	C26—H26	0.9300
Ti1—C13	2.358 (3)	C31—C36	1.381 (4)
Ti1—C11	2.368 (3)	C31—C32	1.386 (4)
Ti1—C14	2.372 (3)	C32—C33	1.375 (5)
Ti1—C15	2.405 (3)	C32—H32	0.9300
N1—C1	1.353 (4)	C33—C34	1.377 (6)
N1—C2	1.386 (4)	C33—H33	0.9300
N2—C1	1.318 (4)	C34—C35	1.368 (5)
N2—C3	1.365 (5)	C34—H34	0.9300
N3—C43	1.465 (5)	C35—C36	1.383 (5)
N3—C41	1.476 (4)	C35—H35	0.9300
N4—C51	1.450 (4)	C36—H36	0.9300
N4—C53	1.460 (5)	C41—C42	1.508 (6)
C1—C4	1.531 (4)	C41—H41A	0.9700
C2—C3	1.351 (5)	C41—H41B	0.9700
C2—H2	0.9300	C42—H42A	0.9600
C3—H3	0.9300	C42—H42B	0.9600
C4—C11	1.536 (4)	C42—H42C	0.9600
C4—C21	1.539 (4)	C43—C44	1.512 (6)
C4—C31	1.547 (4)	C43—H43A	0.9700
C11—C15	1.399 (4)	C43—H43B	0.9700
C11—C12	1.414 (4)	C44—H44A	0.9600
C12—C13	1.385 (4)	C44—H44B	0.9600
C12—H12	0.9300	C44—H44C	0.9600
C13—C14	1.391 (5)	C51—C52	1.508 (5)
C13—H13	0.9300	C51—H51A	0.9700
C14—C15	1.425 (5)	C51—H51B	0.9700
C14—H14	0.9300	C52—H52A	0.9600
C15—H15	0.9300	C52—H52B	0.9600
C21—C26	1.380 (4)	C52—H52C	0.9600
C21—C22	1.388 (5)	C53—C54	1.492 (6)
C22—C23	1.378 (5)	C53—H53A	0.9700
C22—H22	0.9300	C53—H53B	0.9700
C23—C24	1.373 (6)	C54—H54A	0.9600
C23—H23	0.9300	C54—H54B	0.9600
C24—C25	1.353 (6)	C54—H54C	0.9600
			
N3—Ti1—N4	103.96 (13)	C26—C21—C4	122.2 (3)
N3—Ti1—N1	111.06 (10)	C22—C21—C4	120.6 (3)
N4—Ti1—N1	99.38 (10)	C23—C22—C21	121.6 (3)
N3—Ti1—C12	143.42 (14)	C23—C22—H22	119.2
N4—Ti1—C12	104.71 (12)	C21—C22—H22	119.2
N1—Ti1—C12	86.06 (10)	C24—C23—C22	120.1 (4)
N3—Ti1—C13	119.81 (13)	C24—C23—H23	119.9
N4—Ti1—C13	96.74 (13)	C22—C23—H23	119.9
N1—Ti1—C13	120.34 (11)	C25—C24—C23	119.3 (4)
C12—Ti1—C13	34.29 (11)	C25—C24—H24	120.3
N3—Ti1—C11	117.95 (13)	C23—C24—H24	120.3
N4—Ti1—C11	137.59 (11)	C24—C25—C26	120.9 (4)
N1—Ti1—C11	72.40 (10)	C24—C25—H25	119.5
C12—Ti1—C11	34.94 (11)	C26—C25—H25	119.5
C13—Ti1—C11	57.80 (10)	C21—C26—C25	120.8 (4)
N3—Ti1—C14	88.97 (14)	C21—C26—H26	119.6
N4—Ti1—C14	121.12 (12)	C25—C26—H26	119.6
N1—Ti1—C14	129.20 (11)	C36—C31—C32	117.4 (3)
C12—Ti1—C14	56.66 (13)	C36—C31—C4	122.0 (3)
C13—Ti1—C14	34.21 (13)	C32—C31—C4	120.5 (3)
C11—Ti1—C14	57.19 (10)	C33—C32—C31	121.5 (3)
N3—Ti1—C15	87.91 (13)	C33—C32—H32	119.3
N4—Ti1—C15	153.99 (12)	C31—C32—H32	119.3
N1—Ti1—C15	97.66 (10)	C32—C33—C34	120.3 (4)
C12—Ti1—C15	57.06 (12)	C32—C33—H33	119.9
C13—Ti1—C15	57.54 (13)	C34—C33—H33	119.9
C11—Ti1—C15	34.08 (9)	C35—C34—C33	119.1 (4)
C14—Ti1—C15	34.70 (11)	C35—C34—H34	120.4
C1—N1—C2	104.2 (2)	C33—C34—H34	120.4
C1—N1—Ti1	125.6 (2)	C34—C35—C36	120.5 (4)
C2—N1—Ti1	129.7 (2)	C34—C35—H35	119.7
C1—N2—C3	103.8 (3)	C36—C35—H35	119.7
C43—N3—C41	113.4 (3)	C31—C36—C35	121.1 (3)
C43—N3—Ti1	109.1 (2)	C31—C36—H36	119.4
C41—N3—Ti1	137.5 (3)	C35—C36—H36	119.4
C51—N4—C53	113.6 (3)	N3—C41—C42	111.8 (3)
C51—N4—Ti1	128.5 (2)	N3—C41—H41A	109.3
C53—N4—Ti1	117.9 (2)	C42—C41—H41A	109.3
N2—C1—N1	114.1 (3)	N3—C41—H41B	109.3
N2—C1—C4	126.5 (3)	C42—C41—H41B	109.3
N1—C1—C4	118.9 (2)	H41A—C41—H41B	107.9
C3—C2—N1	106.9 (3)	C41—C42—H42A	109.5
C3—C2—H2	126.5	C41—C42—H42B	109.5
N1—C2—H2	126.5	H42A—C42—H42B	109.5
C2—C3—N2	110.9 (3)	C41—C42—H42C	109.5
C2—C3—H3	124.6	H42A—C42—H42C	109.5
N2—C3—H3	124.6	H42B—C42—H42C	109.5
C1—C4—C11	104.4 (2)	N3—C43—C44	114.1 (4)
C1—C4—C21	113.8 (2)	N3—C43—H43A	108.7
C11—C4—C21	109.7 (2)	C44—C43—H43A	108.7
C1—C4—C31	109.3 (2)	N3—C43—H43B	108.7
C11—C4—C31	110.5 (2)	C44—C43—H43B	108.7
C21—C4—C31	109.1 (2)	H43A—C43—H43B	107.6
C15—C11—C12	107.4 (3)	C43—C44—H44A	109.5
C15—C11—C4	129.0 (3)	C43—C44—H44B	109.5
C12—C11—C4	123.3 (3)	H44A—C44—H44B	109.5
C15—C11—Ti1	74.39 (16)	C43—C44—H44C	109.5
C12—C11—Ti1	71.46 (16)	H44A—C44—H44C	109.5
C4—C11—Ti1	115.30 (17)	H44B—C44—H44C	109.5
C13—C12—C11	109.4 (3)	N4—C51—C52	115.1 (3)
C13—C12—Ti1	73.52 (18)	N4—C51—H51A	108.5
C11—C12—Ti1	73.60 (16)	C52—C51—H51A	108.5
C13—C12—H12	125.3	N4—C51—H51B	108.5
C11—C12—H12	125.3	C52—C51—H51B	108.5
Ti1—C12—H12	119.3	H51A—C51—H51B	107.5
C12—C13—C14	107.3 (3)	C51—C52—H52A	109.5
C12—C13—Ti1	72.19 (17)	C51—C52—H52B	109.5
C14—C13—Ti1	73.47 (18)	H52A—C52—H52B	109.5
C12—C13—H13	126.3	C51—C52—H52C	109.5
C14—C13—H13	126.3	H52A—C52—H52C	109.5
Ti1—C13—H13	119.9	H52B—C52—H52C	109.5
C13—C14—C15	109.0 (3)	N4—C53—C54	115.5 (4)
C13—C14—Ti1	72.32 (19)	N4—C53—H53A	108.4
C15—C14—Ti1	73.90 (17)	C54—C53—H53A	108.4
C13—C14—H14	125.5	N4—C53—H53B	108.4
C15—C14—H14	125.5	C54—C53—H53B	108.4
Ti1—C14—H14	120.0	H53A—C53—H53B	107.5
C11—C15—C14	106.9 (3)	C53—C54—H54A	109.5
C11—C15—Ti1	71.53 (16)	C53—C54—H54B	109.5
C14—C15—Ti1	71.40 (18)	H54A—C54—H54B	109.5
C11—C15—H15	126.6	C53—C54—H54C	109.5
C14—C15—H15	126.6	H54A—C54—H54C	109.5
Ti1—C15—H15	122.3	H54B—C54—H54C	109.5
C26—C21—C22	117.2 (3)		
			
N3—Ti1—N1—C1	116.2 (3)	C14—Ti1—C12—C13	37.6 (2)
N4—Ti1—N1—C1	−134.7 (2)	C15—Ti1—C12—C13	79.3 (2)
C12—Ti1—N1—C1	−30.5 (3)	N3—Ti1—C12—C11	−56.3 (2)
C13—Ti1—N1—C1	−31.2 (3)	N4—Ti1—C12—C11	163.17 (17)
C11—Ti1—N1—C1	2.4 (2)	N1—Ti1—C12—C11	64.50 (17)
C14—Ti1—N1—C1	9.4 (3)	C13—Ti1—C12—C11	−116.6 (3)
C15—Ti1—N1—C1	25.5 (3)	C14—Ti1—C12—C11	−79.0 (2)
N3—Ti1—N1—C2	−73.5 (3)	C15—Ti1—C12—C11	−37.26 (16)
N4—Ti1—N1—C2	35.5 (3)	C11—C12—C13—C14	−0.2 (4)
C12—Ti1—N1—C2	139.8 (3)	Ti1—C12—C13—C14	−65.6 (2)
C13—Ti1—N1—C2	139.1 (3)	C11—C12—C13—Ti1	65.4 (2)
C11—Ti1—N1—C2	172.6 (3)	N3—Ti1—C13—C12	−143.4 (2)
C14—Ti1—N1—C2	179.7 (3)	N4—Ti1—C13—C12	106.3 (2)
C15—Ti1—N1—C2	−164.2 (3)	N1—Ti1—C13—C12	1.3 (3)
N4—Ti1—N3—C43	−64.0 (2)	C11—Ti1—C13—C12	−37.2 (2)
N1—Ti1—N3—C43	42.0 (3)	C14—Ti1—C13—C12	−115.0 (3)
C12—Ti1—N3—C43	155.3 (2)	C15—Ti1—C13—C12	−77.8 (2)
C13—Ti1—N3—C43	−170.4 (2)	N3—Ti1—C13—C14	−28.4 (2)
C11—Ti1—N3—C43	122.6 (2)	N4—Ti1—C13—C14	−138.76 (19)
C14—Ti1—N3—C43	174.1 (3)	N1—Ti1—C13—C14	116.21 (19)
C15—Ti1—N3—C43	139.4 (2)	C12—Ti1—C13—C14	115.0 (3)
N4—Ti1—N3—C41	112.6 (4)	C11—Ti1—C13—C14	77.7 (2)
N1—Ti1—N3—C41	−141.4 (3)	C15—Ti1—C13—C14	37.12 (18)
C12—Ti1—N3—C41	−28.1 (4)	C12—C13—C14—C15	−0.6 (4)
C13—Ti1—N3—C41	6.2 (4)	Ti1—C13—C14—C15	−65.3 (2)
C11—Ti1—N3—C41	−60.8 (4)	C12—C13—C14—Ti1	64.7 (2)
C14—Ti1—N3—C41	−9.3 (4)	N3—Ti1—C14—C13	155.6 (2)
C15—Ti1—N3—C41	−44.0 (4)	N4—Ti1—C14—C13	49.9 (2)
N3—Ti1—N4—C51	−9.0 (3)	N1—Ti1—C14—C13	−87.7 (2)
N1—Ti1—N4—C51	−123.6 (3)	C12—Ti1—C14—C13	−37.70 (19)
C12—Ti1—N4—C51	148.1 (3)	C11—Ti1—C14—C13	−79.7 (2)
C13—Ti1—N4—C51	114.1 (3)	C15—Ti1—C14—C13	−116.6 (3)
C11—Ti1—N4—C51	162.3 (2)	N3—Ti1—C14—C15	−87.8 (2)
C14—Ti1—N4—C51	88.4 (3)	N4—Ti1—C14—C15	166.45 (19)
C15—Ti1—N4—C51	106.1 (3)	N1—Ti1—C14—C15	28.9 (3)
N3—Ti1—N4—C53	169.3 (3)	C12—Ti1—C14—C15	78.9 (2)
N1—Ti1—N4—C53	54.7 (3)	C13—Ti1—C14—C15	116.6 (3)
C12—Ti1—N4—C53	−33.6 (3)	C11—Ti1—C14—C15	36.88 (18)
C13—Ti1—N4—C53	−67.6 (3)	C12—C11—C15—C14	−1.2 (3)
C11—Ti1—N4—C53	−19.4 (3)	C4—C11—C15—C14	173.2 (3)
C14—Ti1—N4—C53	−93.3 (3)	Ti1—C11—C15—C14	63.1 (2)
C15—Ti1—N4—C53	−75.6 (3)	C12—C11—C15—Ti1	−64.3 (2)
C3—N2—C1—N1	−0.9 (4)	C4—C11—C15—Ti1	110.2 (3)
C3—N2—C1—C4	−172.4 (3)	C13—C14—C15—C11	1.2 (4)
C2—N1—C1—N2	1.0 (4)	Ti1—C14—C15—C11	−63.2 (2)
Ti1—N1—C1—N2	173.3 (2)	C13—C14—C15—Ti1	64.3 (2)
C2—N1—C1—C4	173.2 (2)	N3—Ti1—C15—C11	−152.97 (19)
Ti1—N1—C1—C4	−14.5 (4)	N4—Ti1—C15—C11	88.6 (3)
C1—N1—C2—C3	−0.6 (4)	N1—Ti1—C15—C11	−42.0 (2)
Ti1—N1—C2—C3	−172.5 (2)	C12—Ti1—C15—C11	38.23 (17)
N1—C2—C3—N2	0.2 (5)	C13—Ti1—C15—C11	79.2 (2)
C1—N2—C3—C2	0.4 (4)	C14—Ti1—C15—C11	115.8 (3)
N2—C1—C4—C11	−168.7 (3)	N3—Ti1—C15—C14	91.2 (2)
N1—C1—C4—C11	20.1 (3)	N4—Ti1—C15—C14	−27.2 (4)
N2—C1—C4—C21	−49.1 (4)	N1—Ti1—C15—C14	−157.8 (2)
N1—C1—C4—C21	139.7 (3)	C12—Ti1—C15—C14	−77.6 (2)
N2—C1—C4—C31	73.1 (4)	C13—Ti1—C15—C14	−36.6 (2)
N1—C1—C4—C31	−98.1 (3)	C11—Ti1—C15—C14	−115.8 (3)
C1—C4—C11—C15	−107.5 (3)	C1—C4—C21—C26	38.4 (4)
C21—C4—C11—C15	130.2 (3)	C11—C4—C21—C26	154.9 (3)
C31—C4—C11—C15	9.9 (4)	C31—C4—C21—C26	−84.0 (3)
C1—C4—C11—C12	66.2 (3)	C1—C4—C21—C22	−144.5 (3)
C21—C4—C11—C12	−56.1 (3)	C11—C4—C21—C22	−28.0 (4)
C31—C4—C11—C12	−176.4 (3)	C31—C4—C21—C22	93.1 (3)
C1—C4—C11—Ti1	−17.5 (3)	C26—C21—C22—C23	1.5 (6)
C21—C4—C11—Ti1	−139.78 (19)	C4—C21—C22—C23	−175.7 (4)
C31—C4—C11—Ti1	99.9 (2)	C21—C22—C23—C24	−2.6 (7)
N3—Ti1—C11—C15	30.9 (2)	C22—C23—C24—C25	2.1 (7)
N4—Ti1—C11—C15	−139.5 (2)	C23—C24—C25—C26	−0.7 (6)
N1—Ti1—C11—C15	135.9 (2)	C22—C21—C26—C25	0.0 (5)
C12—Ti1—C11—C15	−114.9 (3)	C4—C21—C26—C25	177.1 (3)
C13—Ti1—C11—C15	−78.4 (2)	C24—C25—C26—C21	−0.4 (6)
C14—Ti1—C11—C15	−37.6 (2)	C1—C4—C31—C36	5.5 (4)
N3—Ti1—C11—C12	145.87 (18)	C11—C4—C31—C36	−108.9 (3)
N4—Ti1—C11—C12	−24.5 (2)	C21—C4—C31—C36	130.5 (3)
N1—Ti1—C11—C12	−109.15 (18)	C1—C4—C31—C32	−174.7 (3)
C13—Ti1—C11—C12	36.54 (19)	C11—C4—C31—C32	71.0 (3)
C14—Ti1—C11—C12	77.4 (2)	C21—C4—C31—C32	−49.7 (3)
C15—Ti1—C11—C12	114.9 (3)	C36—C31—C32—C33	−0.5 (5)
N3—Ti1—C11—C4	−95.3 (2)	C4—C31—C32—C33	179.6 (3)
N4—Ti1—C11—C4	94.3 (2)	C31—C32—C33—C34	0.4 (6)
N1—Ti1—C11—C4	9.69 (19)	C32—C33—C34—C35	−0.2 (6)
C12—Ti1—C11—C4	118.8 (3)	C33—C34—C35—C36	0.2 (6)
C13—Ti1—C11—C4	155.4 (3)	C32—C31—C36—C35	0.5 (5)
C14—Ti1—C11—C4	−163.8 (3)	C4—C31—C36—C35	−179.6 (3)
C15—Ti1—C11—C4	−126.2 (3)	C34—C35—C36—C31	−0.4 (6)
C15—C11—C12—C13	0.9 (3)	C43—N3—C41—C42	91.0 (4)
C4—C11—C12—C13	−174.0 (3)	Ti1—N3—C41—C42	−85.5 (5)
Ti1—C11—C12—C13	−65.4 (2)	C41—N3—C43—C44	74.6 (4)
C15—C11—C12—Ti1	66.3 (2)	Ti1—N3—C43—C44	−107.9 (4)
C4—C11—C12—Ti1	−108.6 (2)	C53—N4—C51—C52	−62.0 (4)
N3—Ti1—C12—C13	60.3 (3)	Ti1—N4—C51—C52	116.3 (3)
N4—Ti1—C12—C13	−80.2 (2)	C51—N4—C53—C54	−62.7 (5)
N1—Ti1—C12—C13	−178.9 (2)	Ti1—N4—C53—C54	118.7 (4)
C11—Ti1—C12—C13	116.6 (3)		
